# Correlates of Aortic Pulse wave velocity measured by cardiac MRI

**DOI:** 10.1186/1532-429X-13-S1-O97

**Published:** 2011-02-02

**Authors:** Visali Kodali, Yi Wang, Edwin Estrada, Simcha Pollack, Nathaniel Reichek

**Affiliations:** 1 St. Francis Hospital, Roslyn, NY, USA

## Background

Aortic pulse wave velocity (PWV) is often used as an index of aortic compliance(AC). Arterial stiffening and decreased AC with resultant increase in PWV commonly results from aging, hypertension and atherosclerosis. However, correlates of PWV in a large population of carefully screened normal subjects have not been reported.

## Objective

We studied a non-invasive phase contrast cardiac MR (CMR) imaging approach to evaluate PWV and examined its relation to age, body mass index (BMI), coronary CT calcium (CCT) score and other clinical and cardiac variables in a large population of normal and obese subjects.

## Methods

223 consenting nondiabetic, normotensive volunteers without clinical cardiovascular disease or echocardiographic abnormalities (124 females, 116 hyperlipidemic (42 treated), age: 58.6+/-14.9yrs, BMI: 24.8+/-3.3 (24 with BMI>28) were studied. CMR studies were performed on a 1.5 Tesla MR scanner (Siemens, Germany) using a phased array surface-coil and a spine-coil array. PWV was assessed using breath-held through-plane phase-contrast gradient echo imaging of the ascending and descending aorta at the pulmonary artery level with a VENC of 1.5 m/sec, temporal resolution of 24 frames per second and slice thickness of 6mm. Aortic images were manually contoured using QFlow (Medis, Leiden, the Netherlands) and PWV determined by the ratio of the distance between the ascending and descending aortic plane along a candy-cane view over the time of pressure pulse travel in between. Pearson and Spearman correlation coefficients were used to examine associations between PWV and age, CCTscore, blood pressure(BP), rate pressure product(RPP), lipid levels, and cardiac function.

## Results

(Tables 1,2.) Overall and in males and females separately, on univariate analysis, PWV was highly correlated with age, moderately correlated with CCT score and RPP, and had modest correlations with systolic BP. In addition, there were modest correlations of PWV with total cholesterol, triglycerides, HDL and LDL in males and weak correlations with hypertension in females. (All p<0.05) However, after adjustment for age no other variables showed significant association with PWV. Cardiac function parameters including left ventricular(LV) stroke volume, ejection fraction(EF), and mass index have no association to PWV even after adjusting for age and BP and there is no significant difference in PWV between males and females. (p = 0.2).

**Table 1:** Spearman Correlation Coefficients of Aortic Pulse Wave Velocity (1)

	TOTAL POPULATION (n = 223)			FEMALES (n = 124)			MALES (n = 99)		
Mean ± Std deviation	n =	Correlation Coefficients	p-value	n =	Correlation Coefficients	p-value	n =	Correlation Coefficients	p-value
Age 58.6 ± 15.1	221	0.63	<0.0001	123	0.63	<0.0001	98	0.57	<0.0001
Body Mass Index (BMI) 24.8 ± 3.3	168	- 0.002	0.68	91	0.12	0.28	77	0.02	0.85
Hypertension	17	0.17	0.02	11	0.19	0.04	6	0.1	0.32
Systolic Blood Pressure (SBP) 125.3 ± 14.9	221	0.4	<0.0001	124	0.43	<0.0001	97	0.42	<0.0001
Diastolic Blood Pressure (DBP) 72.9 ± 9	221	0.07	0.33	124	0.21	0.02	97	0.06	0.57
Rate-pressure product (RPP) 7957 ± 1637	215	0.36	<0.0001	124	0.33	0.0002	91	0.37	0.0003

**Table 2:** Spearman Correlation Coefficients of Aortic Pulse Wave Velocity (2)

	TOTAL POPULATION (n = 223)			FEMALES (n = 124)			MALES (n = 99)		
Mean ± Std dev	n =	Correlation Coefficients	p-value	n =	Correlation Coefficients	p-value	n =	Correlation Coefficients	p-value

Coronary CT Calcium score (CCT) 122.4 ± 301.3	106	0.46	<0.0001	56	0.54	<0.0001	50	0.32	0.02
Total Cholesterol 204.7 ± 39.6 mg/dl	137	- 0.02	0.8	75	0.08	0.49	62	- 0.27	0.03
Triglycerides 92.4 ± 49.9 mg/dl	137	- 0.03	0.7	75	0.26	0.02	62	- 0.37	0.003
High Density Lipoproteins (HDL) 59 ± 16.9 mg/dl	138	0.15	0.08	75	- 0.09	0.41	63	0.27	0.03
Low Density Lipoproteins (LDL) 126.5 ± 34.3 mg/dl	138	- 0.04	0.6	75	0.12	0.29	63	- 0.28	0.03
Left Ventricular Ejection Fraction (LVEF) 58.7 ± 5.6 %	222	0.11	0.11	123	0.08	0.39	99	0.05	0.63
Left Ventricular Mass index 91.9 ± 24.2gm/m2	221	- 0.02	0.7	123	0.1	0.28	98	0.05	0.66
Left Ventricular Stroke Volume 51.4 ± 9.2 ml	219	- 0.03	0.009	123	- 0.08	0.35	96	- 0.05	0.6

## Conclusions

Age is the strongest and most consistent correlate of increased PWV. CCT score strongly correlates with PWV. After adjustment for age, no other variables show significant associations with PWV. In normal subjects cardiac function parameters do not correlate with PWV.

